# Cooperation between general practitioners, occupational health physicians, and rehabilitation physicians in Germany: what are problems and barriers to cooperation? A qualitative study

**DOI:** 10.1007/s00420-017-1210-6

**Published:** 2017-03-11

**Authors:** Jan M. Stratil, Monika A. Rieger, Susanne Völter-Mahlknecht

**Affiliations:** Institute for Occupational Medicine, Social Medicine and Health Services Research (Institut fuer Arbeitsmedizin, Sozialmedizin und Versorgungsforschung), University Hospital Tuebingen, Wilhelmstrasse 27, 72074 Tuebingen, Germany

**Keywords:** Cooperation, Interface, General practice, Occupational medicine, Rehabilitation, Health services research

## Abstract

**Purpose:**

General practitioners (GPs), occupational health physicians (OPs), and rehabilitation physicians (RPs) fulfill different functions in the rehabilitation process, which need to be interlinked effectively to achieve a successful medical and occupational rehabilitation. In Germany, this cooperation at the interfaces is often suboptimal. The aim of this study was to identify and discuss perceived barriers to cooperation between GPs, OPs, and RPs.

**Methods:**

We used a qualitative study design with eight focus group discussions (FGD) with GPs, OPs, RPs, and rehabilitants. Two FGDs per expert group with 4–10 participants were conducted. The transcripts were analyzed using qualitative content analysis.

**Results:**

A number of obstacles to cooperation were reported by the participants, including (1) organizational (e.g., missing contact details, low reachability, schedule restrictions), (2) interpersonal (e.g., rehabilitants level of trust in OPs, low perceived need to cooperate with OPs, low motivation to cooperate), and (3) structural barriers (e.g., data privacy regulations, regulations concerning rehabilitation reports).

**Conclusion:**

The present data agree with study results from other countries, which addressed interfaces in the rehabilitation process. While some barriers could be overcome by the participants themselves, a multi-level stakeholder approach might be necessary. Future quantitative research is required to assess the relative weight of the findings.

## Background

General practitioners (GPs), occupational health physicians (OPs), and rehabilitation physicians (RPs) fulfill different functions in the rehabilitation process. These need to be interlinked effectively to achieve successful medical and occupational rehabilitation. In Germany, these interfaces have been criticized for many years as suboptimal. In particular, it is criticized that the involvement of OPs in the process is not sufficient (Dasinger et al. 2001). This finding was confirmed by two recent international literature reviews on the cooperation between OPs and RPs (Rijkenberg et al. 2013; Völter-Mahlknecht and Rieger 2014) as well as one review on the cooperation between GPs and OPs (Mosshammer et al. 2011).

The cooperation and communication of OPs and RPs have been investigated in a number of surveys involving RPs, OPs, and rehabilitants from Austria, the Netherlands, Belgium, and Germany (van Amstel and Buijs 2000; Seidel et al. 2003; Luedemann 2006; Vroeijenstijn-Nguyen and Brenner 2007; Rijkenberg 2012; Mueller et al. 2013). Although OPs and RPs expressed an interest in improving communication and cooperation (van Amstel and Buijs 2000; Seidel et al. 2003; Vroeijenstijn-Nguyen and Brenner 2007; Rijkenberg 2012; Mueller et al. 2013), these studies found a low intensity of communication and cooperation between OPs and RPs in all four countries. Especially, the studies from Germany indicated an exclusion of OPs from the rehabilitation process (Seidel et al. 2003; Tavs 2005; Luedemann 2006; Mueller et al. 2013). Other studies underlined the survey findings, e.g., by stating that there is no regular, systematic communication between RPs and OPs (Schwarze et al. 2013), that OPs often receive information on their rehabilitants’ rehabilitation treatment months after the discharge or not at all (Manecke et al. 2008).

A general need for improvement of the cooperation between OPs and GPs, including in the field of rehabilitation was concluded by studies from Germany and the UK (Beaumont 2003; Beach and Watt 2003; Mosshammer et al. 2011, 2016). However, the cooperation and communication between GPs and OPs concerning rehabilitation have not yet been investigated intensively.

OPs, GPs, and RPs agree that an efficient interaction between the protagonists is necessary for successful rehabilitation and occupational reintegration, and that cooperation and communication need to be strengthened (de Bono 1997; Buijs et al. 1999; van Amstel and Buijs 2000; Jakobsson et al. 2002; Schochat et al. 2003; Beaumont 2003; Rijkenberg 2012).

A number of international literature reviews analyze interventions which improve the work-related health of rehabilitants (e.g., in regard to reduced sick leave and time to first return to work). These include individualized rehabilitation according to need and capacity for a specific workplace, work accommodations (e.g., ergonomic improvements), early contact of the worker to the workplace, and contact of the health care provider with the rehabilitant’s workplace (Franche et al. 2005; Steenstra et al. 2006; Bethge and Mueller-Fahrnow 2008; Carroll et al. 2010; van Vilsteren et al. 2015). Most of these aspects lie within the responsibility of OPs.

For the setting of the German rehabilitation process, studies have indicated that improved cooperation in the rehabilitation process and especially the inclusion of OPs is beneficial in improving the occupational health of patients (Trowitzsch and Rust 2000; Kuehn et al. 2008; Mueller et al. 2009; Schwarze et al. 2013; Bethge et al. 2016).

The German code of social law differentiates between medical, occupational, and social rehabilitation. A patient is eligible for medical rehabilitation if the patient’s earning capacity is substantially at risk or already diminished. In these cases, the funding agency will be the German Pension Found (DRV) (BAR 2005; Hallier et al. 2013). Medical rehabilitation in Germany includes the treatment by physicians, physical and/or psychological therapy, stress tests and occupation-focused rehabilitation therapy (*MBOR*) as well as the provision of assisting devices and step-wise (occupational) reintegration (§ 15 SGB VI, §§ 26–31 SGB IX).

Rehabilitation therapy is initiated by patients by filing an application, which needs to include a health assessment report by a GP, OP, or another medical specialist (BAR 2005; Hallier et al. 2013). In the federal state of Baden-Wuerttemberg, OPs can initiate and coordinate an OP-guided rehabilitation (*B.Ä.R*.) (DRV Baden-Wuerttemberg). The funding agencies assess and decide on the patients’ applications and if it is rejected, an objection can be filed. At the end of rehabilitation therapy, the rehabilitation institution should assess the need of occupational reintegration. A proposed plan for occupational reintegration needs to be approved by the rehabilitant, the treating physician, and the employer.

After rehabilitation, the physicians treating the patients (e.g., GP) are informed by the RP via a rehabilitation report and/or a short physician’s letter. In the post-rehabilitation phase, GPs plan and organize the follow-up treatment and are involved in occupational reintegration of the patients (BAR 2005). The OPs’ role includes assessing, preparing, and discussing options for the patients’ occupational reintegration. To facilitate the reintegration process OPs can manage the provision of work accommodation (e.g., assisting devices) as well as determine the need and possibilities for retraining and job rotation (Leitner et al. 2009; Panter 2012).

The aim of this study was to identify and discuss barriers to cooperation between RPs, OPs, and GPs. Based on the literature (Rijkenberg et al. 2013; Völter-Mahlknecht and Rieger 2014), this article focuses on the role of OPs at these interfaces. In particular, we aim to answer—amongst others—the following questions: (1) How do the medical stakeholders and rehabilitants experience and evaluate the cooperation and communication in terms of quality and intensity? (2) What barriers and obstacles to cooperation and communication do the participants perceive and experience?

## Methods

We conducted an explorative qualitative study based on eight Focus Group Discussions (FGDs) and used qualitative content analysis for data analysis. The questions in the FGDs focused on (1) attitudes towards rehabilitation therapy (warm-up question), (2) the perceived role and function of OPs, GPs, and RPs in the rehabilitation process, (3) the informational need of patients and medical stakeholders, and (4) the experienced quality and intensity of cooperation and communication at the interfaces. The full interview guide will be provided by the authors upon request.

### Study population

Two FGDs were conducted each with rehabilitants (7 and 7 participants per FGD) as well as the main medical stakeholders: GPs (6 and 10), RPs (6 and 6), and OPs (4 and 5). The composition of participants followed the principle of maximal structural variation (Patton 1990) to represent the heterogeneity of protagonists in the field. The study sample is shown in Table 1. OPs were recruited via telephone from members of the Association of German Occupational and Company Physicians (Verband Deutscher Betriebs- und Werksärzte (VDBW)). RPs and patients were recruited through cooperation with the rehabilitation clinics Treatment Center Federsee (Therapiezentrum Federsee) in Bad Buchau (specializing inter alia in orthopedic medicine, oncology, and rheumatology) and the Huettenbuehl clinic of the Rehabilitation Center Bad Duerrheim (Reha-Zentrum Bad Duerrheim, Klinik Huettenbuehl) in Bad Duerrheim (specializing in alia in psychosomatic illnesses and mental health). GPs were recruited via E-mail from medical practices associated with the Department for General Medicine at the Medical Faculty of the University of Tuebingen. An incentive of 50 € for physicians and 30 € for patients was offered.

**Table 1:** Characteristics of focus group participants

Physicians	General practitioners	Occupational physicians	Rehabilitation physicians	Rehabilitants	Rehabilitants
Participants	*n* = 22	*n* = 9	*n* = 12	*n* = 15	Participants
Age average [median/(range)]	57/(40–67) years	55/(45–65) years	48/(34–58) years	53/(22–63) years	Age [median/(range)]
Sex: nbr. female	*n* = 9	*n* = 5	*n* = 6	*n* = 8	Sex: nbr- fem
Work experience as physician	27/(13–40) years	29/(12–39) years	13/(6–30) years	One: *n* = 4 Two: *n* = 1 Three: *n* = 1	Previous rehabilitation therapies
Work experience in specialization [median/(range)]	21/(7–33) years	20/(1–32) years	11/(3–31) years		
Type of employment	Solo practice: *n* = 13 Group practice: *n* = 9	Employed at one/several enterprise *n* = 1 Employed in Occupational health service for one/several enterprises: *n* = 4 Freelance for one/several enterprises: *n* = 4		21 days: *n* = 4 28 days: *n* = 3 35 days: *n* = 5 >35 days: *n* = 3	Planned duration of rehabilitation (days)
Practice site	Urban: *n* = 2 Rural: *n* = 10 Mixed: *n* = 10	Urban: *n* = 5 Rural: *n* = 0 Mixed: *n* = 4		Mental health *n* = 5 Musculoskeletal *n* = 5	Reason for rehabilitation
Practice size (patients per 3 months)	<700: *n* = 2 700–1400: *n* = 14 > 400: *n* = 5	Responsible for SME: *n* = 8		Office work: *n* = 5 Industrial production: *n* = 3 Construction work: *n* = 1 Logistic sector: *n* = 1 Nursing care: *n* = 2 Pedagogue: *n* = 1 Cleaner: *n* = 1	Occupation
Rehabilitation applications [median/(range)]	35/(5–50) per Year				
				Small or medium enterprises: *n* = 7	Type of employer
Within catchment area of a company medical service?	In town: *n* = 7 In the country: *n* = 3 Both: *n* = 2 Without: *n* = 8			Business has OP: *n* = 8 Patient knows OP: *n* = 7	Relationship to OP (responses by patients)

### Focus group discussions

FGDs are an established method of data collection in qualitative research (Liamputtong 2013; Krueger and Casey 2014). Supported by guiding questions, the participants engage in an in-depth discussion of various topics (Morgan and Spanish 1984; Kitzinger 1994). The semi-structured FGDs were conducted between February and May 2015 (duration: 85–99 min) by two female researchers working for the Institute of Occupational Medicine, Social Medicine and Health Services Research at the University of Tuebingen. Three OPs and one GP were already acquainted with one interviewer. The participants were informed on the professional background of the interviewers and the aim of the research project prior to the discussions. Both GP-FGDs and one OP-FGD took place at the University Hospital of Tuebingen resp. in our institute in Tuebingen. The other OP-FGD was held in a conference room in Stuttgart, which was closer to the participants, and the FGDs with RPs and patients were conducted in the rehabilitation clinics.

### Data analysis

We used qualitative content analysis (Mayring 2014) and the software MAXQDA 11 (VERBI GmbH; Berlin, Germany) for data analysis. First, the audio files were transcribed and pseudonymized. We went through the transcripts line by line and built inductive categories from the material. Step by step, passages were either subsumed under categories already built or a new category was formulated. After working through three out of the eight transcripts, we assumed that saturation was reached as no new categories could be identified. At this point, we revised the coding frame and assessed whether it met the research questions. Next, we applied the categories deductively on the complete set of all eight transcripts (Mayring 2014). Throughout the whole process, two to three (neutral) persons worked partly independently from each other on the same steps, partly in close discussion. This was done in order to fully exploit the richness of the data, to control for subjective blurring, and to achieve intersubjective certifiability by including and discussing multiple perspectives in the research process (Lucius-Hoene and Deppermann 2004). Content validation was carried out in a workshop in January 2015. Representatives of all parties were invited with a total of 16 GPs and OPs participating.

## Results

### Category system

We identified four main categories: (I) “perceived interfaces between the protagonists,” (II) “perceived problems in the rehabilitation process,” (III) “perceptions of and attitudes towards the own group and other stakeholders,” and (IV) “perceived role of protagonists in the rehabilitation process”.

The first main category (I) “perceived interfaces between the protagonists” included the categories (I.a) “Interfaces between protagonists in general,” (I.b) “prior…,” (I.c) “during…,” and (I.d) “after & at the end of rehabilitation treatment.” Each of these four categories consists of the subcategories “type of interface” and “quality and intensity of cooperation & communication,” The fifth category in the main category (I) was (I.e) “Barriers to cooperation.”

The second main category (II) “perceived problems in the rehabilitation process” consists of the categories (II.a) “prior…,” (II.b) “during…” and (II.c) “after & at the end of rehabilitation therapy.” Further categories in the main category (II) were (II.d) “concerning application process,” (II.e) “concerning the rehabilitation report & the short letter,” (II.f) “concerning small- & medium-sized enterprises (SMEs),” as well as (II.g) “issues of data privacy.”

### Cooperation and communication between the protagonists

**Fig. 1:**
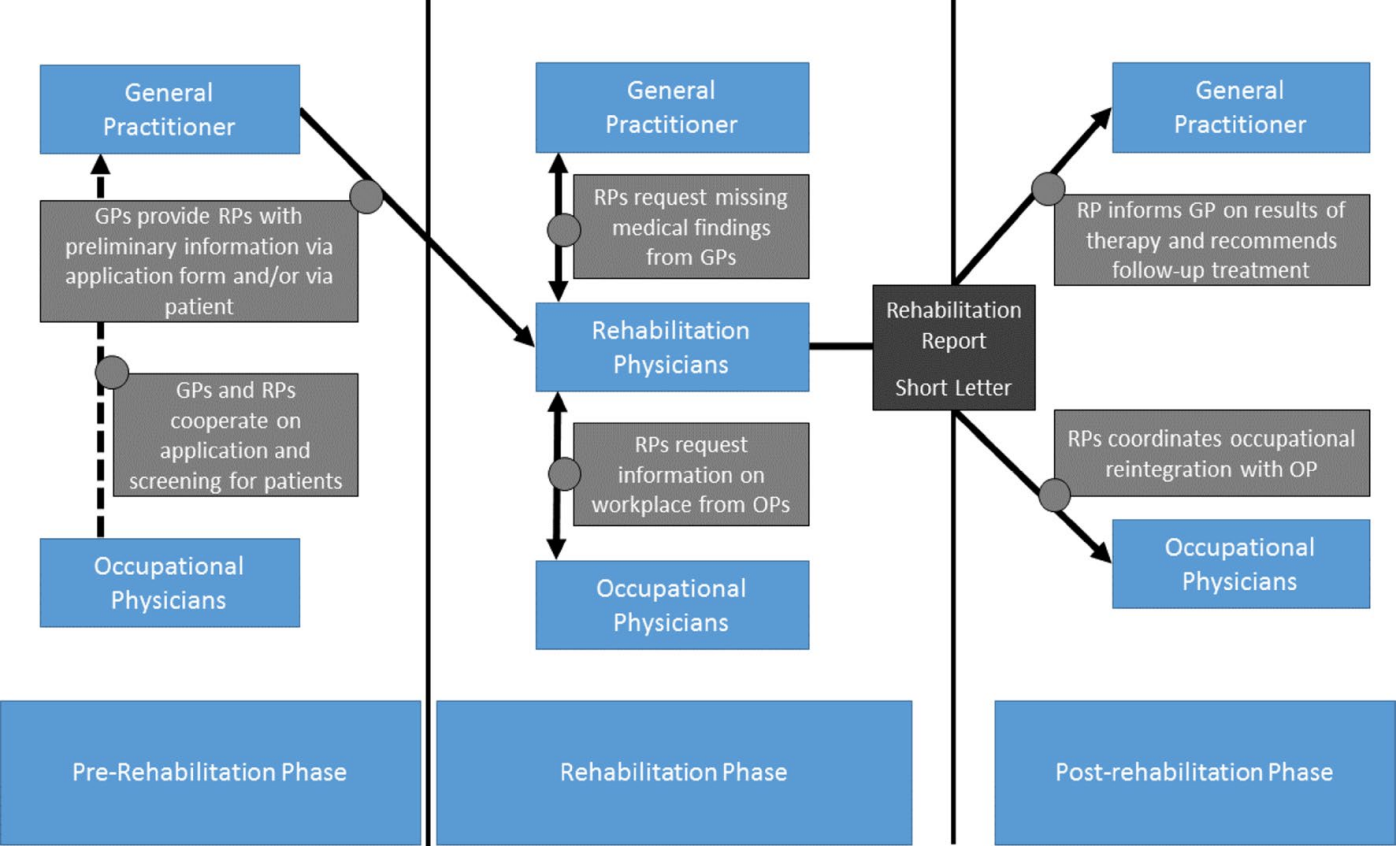
Interfaces in the different stages of rehabilitation process between OPs, RPs, and GPs as reported by the participants in our study

First, we will outline how the participants perceived and experienced the cooperation and communication at their interfaces at the beginning, during, and at the end of rehabilitation in regard to the type of interface as well as the quality and intensity of communication and cooperation. This is displayed in Fig. 1.

#### Cooperation and communication at the interfaces in general

The cooperation with OPs was described as low in intensity by all participants and OPs criticized being left out of the rehabilitation process. OPs stated they often did not receive information directly from the rehabilitation clinics. Further, they reported sometimes learning about a patient’s rehabilitation therapy weeks or months after discharge. While all OPs, most RPs, and some GPs stated that they wished to improve cooperation with OPs, some GPs and RPs as well as most patients were more hesitant.

The RPs mentioned GPs as their main cooperation partners in the rehabilitation process and characterized their cooperation as working well in general with need for improvement concerning the transmission of preliminary medical findings. In contrast, a number of GPs stated having little to no contact with RPs aside from the rehabilitation report and characterized the interface as functioning poorly. Most patients in the FDGs had little knowledge about the intensity and quality of cooperation at the interfaces. The rehabilitation process of two patients was initiated by an OP. These patients perceived that the cooperation between RPs and OPs was working well.

### Interfaces prior to rehabilitation treatment

GPs provide RPs with preliminary medical findings prior to rehabilitation therapy via the application form or the patients themselves. According to RPs, documents were often missing and had to be requested from the GP, which resulted in a delay in assessment and treatment. Despite all four groups of stakeholders regarded a cooperation of GPs and OPs on screening and the application process as desirable and useful, none of our participants reported that such cooperation already existed.

#### Interfaces during the rehabilitation treatment

Providing information on the patient’s workplace upon request of the RPs was reported to be a main interface between RPs and OPs. Most OPs reported that they were rarely asked to provide this information, while RPs reported rarely relying on the OPs assessments. Most OPs perceived this low flow of information as problematic as a sole reliance on subjective statements of patients was prone to bias. In regard to employees of larger companies RPs stated that this interface was working well. However, requesting workplace descriptions for patients from small and medium-sized enterprises (SME) was described as arduous and often not leading to the expected results.

#### Interfaces after and at the end of the rehabilitation treatment

RPs providing GPs, OPs, or medical specialists with information needed for the follow-up treatment was considered the main interface at the end of rehabilitation. This interface was mainly and often exclusively established through the short physician’s letter and the rehabilitation report, according to all physicians.

Most OPs criticized that they rarely received the rehabilitation report directly from the rehabilitation clinics, even if the patient had given his/her consent and that OPs were rarely integrated in the occupational reintegration process, especially in SMEs. This is in accordance with statements of RPs that they did not consider OPs to be obligatory recipients of the rehabilitation report and felt that integration of OPs was rarely necessary. Most patients were not aware of the OP’s role in the occupational reintegration and of OPs as possible recipients of the rehabilitation report.

In the federal state of Baden-Wuerttemberg, OPs can initiate rehabilitation therapy as part of a structured OP-led rehabilitation process (*B.Ä.R*.). In general, OPs perceived this program as successful, although employees of SMEs rarely benefitted from it. Two patients reported to have made positive experiences with *B.Ä.R*. Patients, OPs, and one RP stated that communication in *B.Ä.R*. was working well.

### Barriers to cooperation

Next, we will outline the organizational, interpersonal, and structural barriers to cooperation and communication found by means of the FGDs, as displayed in Table 2 and Fig. 1. Organizational barriers refer to practical barriers, which arose in the working routine of the stakeholders. Interpersonal barriers refer to obstacles, which the participants ascribed to the role, character, or interests of stakeholders. Structural barriers refer to barriers, which were perceived as being caused by regulations and the structure of the system the protagonists are placed in.

**Table 2:** Barriers to cooperation between OPs, RPs, and GPs during the rehabilitation process found in our study with quotations from the interviews

Subcategories	Quotations
Organizational barriers	
Missing contact details of OPs	F2: “…when we’re dealing with small companies that only see the OP once or twice a year, then [contacting the OP] is practically impossible.” (RP II,127–130)
Low reachability of RPs, OPs, and GPs	M1: “…today I contacted a company physician and it took five phone calls until I had him on the line. He’s only there Tuesdays and Thursdays and only at this and that time. Than that has to fit into my schedule.” (RP I, 62)
Time restriction of RPs and GPs	M1: “[It would be helpful] to facilitate the flow of information to occupational or company physicians […], but at the moment I have no real idea how we could manage this in our daily routines.” (RP II 83)
Need for fast coordination on short notice	M1: “Naturally [coordination with OPs regarding occupational reintegration] must happen in a timely manner… [Recommendations can only be made during the course of rehabilitation therapy]. And then it needs to be quick, then you can’t say something like: okay, you’ll get your answer in ten days-… That needs to be done within two or three working days.” (RP II, 303)
Interpersonal barriers	
Relationship between patients and OPs and level of trust of patients	F2: “But to the company physicians, there’s hardly any contact, if any. And that has a lot to do, speaking from my own experience here, a lot to do with prejudices and fears [of the rehabilitants] that confidentiality will be neglected with regard to their employers, etc.” (RP I, 40)
Low perceived need to cooperate with OPs	M4: “…[with regard to workplace assessments] you usually reach a reasonable result in, up to 90 percent [of patients]. In rare cases, the occupational health physician or company physician actually does send us some kind of protocol from the workplace. […] Usually there are hardly any problems [in the assessment without input from OPs].” (RP II, 96–98)
Lacking initiative of RPs, OPs, or GPs	F2: “In the 18 years [in which I’ve worked as a GP], I have never had contact with an OP, I don’t know what they do […] M5: “The fact is that contact is made primarily through our own private initiatives, and usually ends negatively.” (GP I, 364–367)
Structural barriers	
Structure and length of rehabilitation report	M3: “[If we would call the GPs more often and talk on the phone], more information would be conveyed naturally than in just a report. Aside from that, as I mentioned, we are unfortunately formally obliged to formulate eight to ten-page reports that, as a rule, the physicians don’t even read or only read small parts of.” (RP I, 191)
Data privacy regulations	F4: “If we could write an E-Mail now, […] I believe that would be more helpful, if they could chose the time when to read this information themselves.” M1: “And that’s where the data privacy regulations of the German pension insurance do not take effect. Because we still don’t have a secure E-Mail system. Right now we’re required to refrain from sending any E-mails with patient data to anyone, not even to the GP.” (RP I, 279–280)
Different usage of terms for ability to work	Interviewer: > “…it is often difficult for the rehabilitant that they say their GP tells them something different than the rehabilitation physician. My OP says something completely different. Each has their own philosophy about what I can do, … my state of health.” W2: “But that sometimes depends on these differences in language use.” (OP I, 188–193)
Small- and medium-sized enterprises	M1: “Workplace descriptions are available for large companies. There are no descriptions for small and medium -sized enterprises, or only to a limited extent” (RP II, 95)

In brackets: section in the transcript. In bold: pseudonymization codes of the interview partners (F: female participant, M: male participant)

#### Organizational barriers

According to RPs, missing contact details posed a barrier to cooperation with OPs. This information was often missing in the application and could not be provided by patients. The latter was supported by the interviewed patients, as a considerable number of patients did not know the OP responsible for them.

Low reachability of OPs and RPs was mentioned as barriers by all groups of physicians. GPs and OPs both perceived contacting RPs as cumbersome and complicated due to a low reachability and unclear responsibilities within the rehabilitation clinic.

Time restrictions on the part of the GP were perceived as a barrier to communication by RPs, e.g., GPs often had no time to discuss individual cases. GPs also perceived time restrictions as a barrier to communication with OPs and RPs, but associated these deficits in cooperation with shorter working hours of OPs and RPs in comparison with GPs.

RPs stated that coordination with OPs concerning occupational reintegration was complicated because coordination needed to materialize quickly and on short-notice. The assessment of the patients’ needs for occupational reintegration is made at a late stage of rehabilitation therapy. Therefore, communication with OPs and feedback needed to be completed within few working days, which was often not regarded as feasible.

The patients seemed to have little knowledge about the organizational barriers at the interfaces.

#### Interpersonal barriers

A low need for cooperation with OPs was mentioned by RPs. While OPs perceived an external workplace description as important for successful rehabilitations, RPs felt they were able to sufficiently assess the patients’ workplaces and therefore rarely requested information. The patients believed to be able to sufficiently inform RPs on their workplace.

Some RPs believed the integration of OPs into the occupational reintegration process as rarely necessary and considered OPs to be optional recipients of the rehabilitation report. OPs in both FGDs attributed their experience of being left out of the rehabilitation process to an insufficient knowledge of GPs and RPs of the OPs functions and capabilities. The OPs’ perceptions were supported by one RP who was not aware that OPs were involved in occupational reintegration at all. Similar statements were made in one GP-FGD and both patient-FGDs. They responded that they were not aware of the OPs’ function in general and therefore did not know of the OPs’ role in the rehabilitation process.

Lacking initiative on part of OPs and RPs was reported in both GP-FGPs to pose a barrier to cooperation. Some GPs reported never or hardly ever having experienced an OP trying to contact them. Similar experiences were reported by RPs and OPs about the other groups of physicians. RPs and OPs both experienced that the cooperation was greatly improved when physicians on either side were committed to OP-RP-collaboration.

RPs and OPs both stated that patients’ concerns often posed a barrier to cooperation between these protagonists. Some rehabilitants would not allow RPs to contact OPs (e.g., to request information). Moreover, patients demanded to decide if OPs should receive the rehabilitation report and thereby whether they were included in the reintegration process. The patients’ demands were supported by RPs and GPs alike. Therefore, the relationship and trust between patients and OPs were considered important for the cooperation between RPs and OPs. In the interviews, the majority of patients reported either not to know or not to trust their OPs. Some feared that the OP might inform the employer about their condition. This aligns with RPs’ experiences. In contrast, two patients reported having a good and trustful relationship with their OP. Some OPs attributed these attitudes to an insufficient knowledge about the OPs’ medical confidentiality.

#### Structural barriers

Data privacy regulations posed an obstacle to cooperation between RPs and OPs according to these protagonists. The patient’s approval is needed for direct communication between RPs and OPs and also for OPs to receive the rehabilitation report. According to RPs, data privacy regulations in Germany prohibit the use of E-mails as long as no proper encryption was made available by the DRV.

Structure and length of the rehabilitation report were repeatedly reported as posing a problem by OPs, RPs, and GPs. It was perceived as too long and often containing unnecessary information. According to RPs, this led to recipients not to read the report as a whole and to miss relevant information. GPs argued that a leaner report would also allow shorter delivery times. Some RPs did not consider the length of the report to be an issue and argued that the comprehensiveness of the information might be needed by specialists. Length and structure were attributed to regulations set by the funding agencies.

According to OPs, RPs, and GPs, differing assessment of the patient’s working ability posed obstacles at the interfaces. In the worst case, this could lead to the patient losing his/her job. GPs attributed the differences to funding agencies’ regulations, which incentivized RPs to discharge patients in a status able to work. RPs ascribed the differing assessments to GPs not reading the whole rehabilitation report, a superficial knowledge of the patients’ occupations, and being unfamiliar with legal definitions. OPs attributed these differences in the assessment to a different understanding of key terms (e.g., of the term *piecework*) and an insufficient knowledge of RPs on the patients’ workplaces. Some patients had received contradicting information concerning their ability to work by different physicians.

Collaboration with OPs was regarded as complicated by RPs when the rehabilitant worked in SMEs as obtaining workplace information was time-consuming and the reachability of OPs was lower. These experienced issues with SMEs are in accordance with statements from OPs and rehabilitants.

## Discussion

**Fig. 2:**
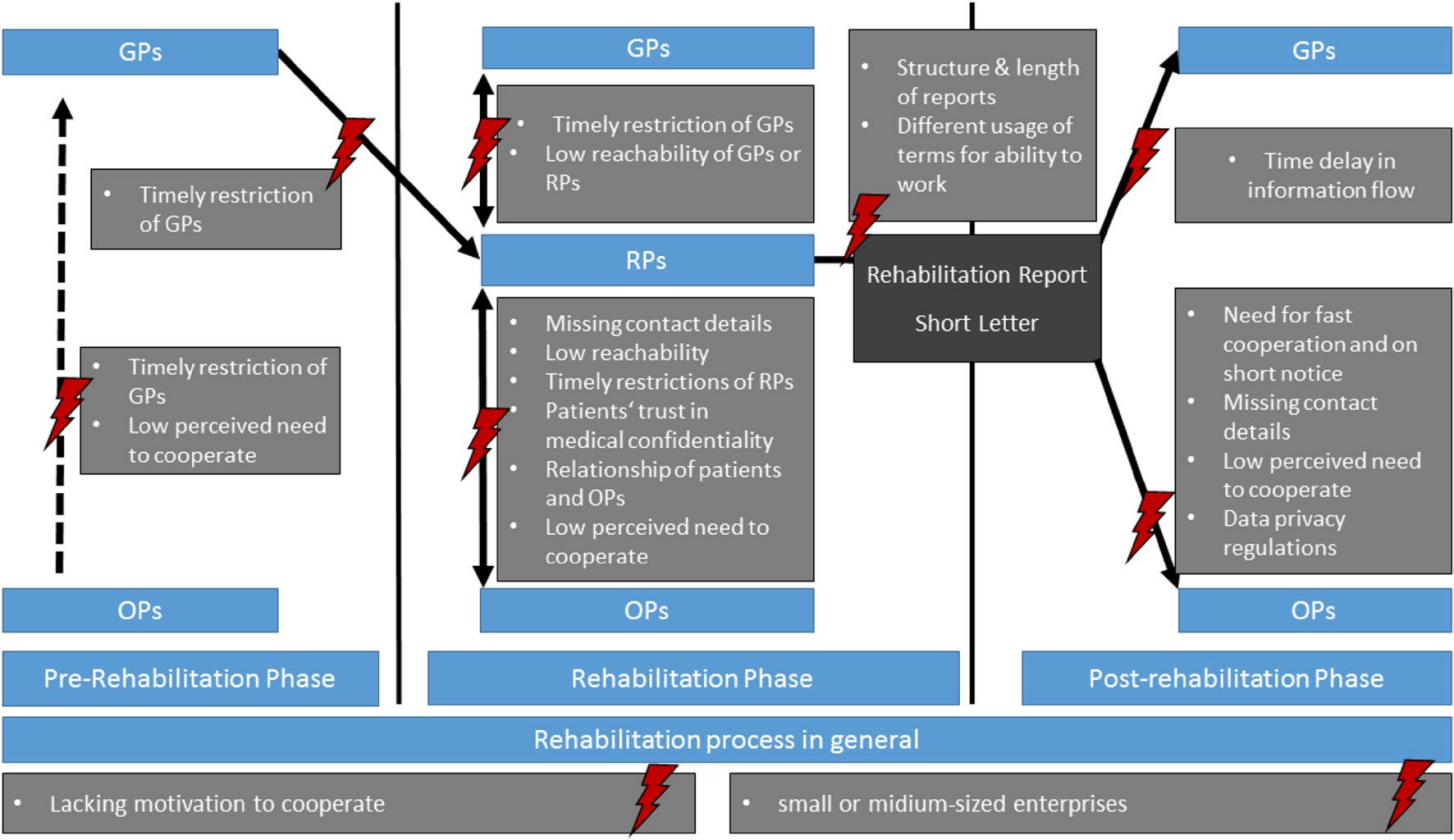
Barriers to cooperation and communication at the interfaces in the rehabilitation process as mentioned by GPs, RPs, Ops, or patients

Participants in this qualitative study perceived the cooperation between GPs, RPs, and OPs in the rehabilitation process as not working smoothly. Especially OPs felt excluded from the process. RPs, OPs, GPs, and rehabilitants reported a number of obstacles to cooperation, including organizational, interpersonal, and structural barriers. These barriers are described in Fig. 2. While the nature of the method used does not allow conclusions concerning the representativeness of issues highlighted by the participants, our findings are in line with studies conducted in Germany and Western Europe.

The low levels of integration of OPs are in accordance with a number of German publications (Rijkenberg et al. 2013; Völter-Mahlknecht and Rieger 2014).

The organizational barrier of a lack of time and the reachability of communication partners were mentioned by OPs and RPs from the Netherlands, Belgium, and Austria (Vroeijenstijn-Nguyen and Brenner 2007; Rijkenberg 2012). These issues were also addressed as barriers to the cooperation between OPs and other medical specialists (Mosshammer et al. 2011).

As an interpersonal barrier, we found that the withholding of contact approval by patients could pose a barrier to cooperation between RPs and OPs. This finding is supported by a survey of German rehabilitants (Luedemann 2006) and RPs from Austria and Belgium (Rijkenberg 2012).

The interpersonal barrier of RPs and GPs being unaware of the OPs’ role and function in the rehabilitation process was reported in studies from Germany, the Netherlands, Belgium, and Austria (van Amstel and Buijs 2000; Rijkenberg 2012; Mueller et al. 2013; Mosshammer et al. 2012).

GPs’ mistrust of OPs was reported in studies from Germany, the Netherlands, Belgium, and the UK, e.g., in terms of OPs not working in the interest of the patient and not sticking to confidentiality regulations (Buijs et al. 1999; Nauta and von Grumbkow 2001; Pfaff et al. 2009; Mosshammer et al. 2011). Two Dutch surveys found similar perceptions among RPs (van Amstel and Buijs 2000; Vroeijenstijn-Nguyen and Brenner 2007).

A strength of the study is that not only physicians, but also rehabilitants were involved as main stakeholders. We were also able to attain a nearly optimal heterogeneity in the FGDs of GPs, RPs, and rehabilitants (e.g., on the characteristics: sex, working experience, company size of OPs, disease patterns). As the recruitment of OPs turned out to be complicated, a selection bias of OPs with a strong interest in the topic cannot be ruled out. Consequently, the actual composition of our focus groups deviates from the planned composition, especially concerning the OPs. As some OPs had worked as employees of occupational service providers and as staff doctors in the past, we believe their perspectives is represented in our interviews as well. As some studies indicate a strong heterogeneity of rehabilitation clinics, a bias in the RPs and rehabilitants perception due to unwanted group effects cannot be precluded. As the study was conducted by occupational health experts, biased responses due to social desirability are possible, but it can be considered low due to the richness of our data and the critical statement made in the discussions.

Our study provides an overview of barriers to the cooperation perceived by German GPs, RPs, OPs, and rehabilitation patients. The main problem area related to organizational, interpersonal, and structural barriers. As discussed, the presented data generally align with the results of studies from other European countries. Future quantitative research is required to better assess the weight of the suggestions presented here.

Some of the barriers could be overcome by the protagonists themselves or by regional cooperation in the current milieu. Other barriers will require interventions in the areas of finance, data regulation, and the rehabilitation report requirements. Therefore, it seems that ongoing interventions on various levels and by different stakeholders might be necessary, including state and federal actors.

We suggest focusing on the organizational and interpersonal barriers, as these might be easier to overcome by the stakeholders themselves. OPs should focus on how they can foster trust of employees in the medical confidentiality and on how to deepen doctor-patient relationships. Also, OPs should focus on informing GPs and RPs on the mutual benefits of strengthening cooperation. One opportunity could lie in joint continuing medical education programs. Furthermore, top-down interventions could focus on strengthening the role of OPs in the rehabilitation process, e.g., by making the contact details or information on the workplace an obligatory part of the application form.
